# Nasopharyngeal Pneumococcal Density and Evolution of Acute Respiratory Illnesses in Young Children, Peru, 2009–2011

**DOI:** 10.3201/eid2211.160902

**Published:** 2016-11

**Authors:** Roger R. Fan, Leigh M. Howard, Marie R. Griffin, Kathryn M. Edwards, Yuwei Zhu, John V. Williams, Jorge E. Vidal, Keith P. Klugman, Ana I. Gil, Claudio F. Lanata, Carlos G. Grijalva

**Affiliations:** Vanderbilt University, Nashville, Tennessee, USA (R.R. Fan, L.M. Howard, M.R. Griffin, K.M. Edwards, Y. Zhu, C.F. Lanata, C.G. Grijalva);; University of Pittsburgh, Pittsburgh, Pennsylvania, USA (J.V. Williams);; Emory University, Atlanta, Georgia, USA (J.E. Vidal, K.P. Klugman);; Instituto de Investigacion Nutricional, Lima, Peru (A.I. Gil, C.F. Lanata)

**Keywords:** Streptococcus pneumoniae, pneumococcal pneumonia, pneumococcal infections, pneumococcal density, nasopharynx, bacterial load, respiratory tract infections, acute respiratory illnesses, young children, Peru, bacteria

## Abstract

We examined nasopharyngeal pneumococcal colonization density patterns surrounding acute respiratory illnesses (ARI) in young children in Peru. Pneumococcal densities were dynamic, gradually increasing leading up to an ARI, peaking during the ARI, and decreasing after the ARI. Rhinovirus co-infection was associated with higher pneumococcal densities.

*Streptococcus pneumoniae* commonly colonizes the nasopharynx of young children (*1*). Nasopharyngeal colonization density is relevant for transmission of bacteria and pathogenesis of pneumococcal diseases (*2*). Few studies have evaluated the longitudinal relationship between nasopharyngeal pneumococcal density and acute respiratory illnesses (ARIs). We examined the evolution of nasopharyngeal pneumococcal density surrounding ARIs in young children.

## The Study

We performed sequential cross-sectional assessments from a prospective cohort study of Andean children in Peru (*3*). During 2009–2011, children <3 years of age from the District of San Marcos, Cajamarca, Peru, were assessed for ARIs during weekly household visits. The population was rural and had low incomes and limited access to healthcare (*3*,*4*). Use of 7-valent pneumococcal conjugate vaccine (PCV7) started in late 2009. Institutional review boards of Vanderbilt University (Nashville, TN, USA) and the Instituto de Investigacion Nutricional (Lima, Peru) approved the study.

An ARI episode was defined as the length of time a child had cough or fever (*5*,*6*). If a child was ill during a household visit, we assessed for pneumonia or lower respiratory tract infection using IMCI-WHO (Integrated Management of Childhood Illness–World Health Organization) criteria (*5*,*7*). If the child had an ARI during the preceding 7 days, we collected a nasal swab sample and tested it for respiratory viruses by reverse transcription PCR at Vanderbilt University (*6*,*8*–*11*). Nasopharyngeal swab samples were collected monthly without regard to ARI and tested at Emory University (Atlanta, GA, USA) by using quantitative PCR for pneumococcal density determinations. For this study, we used samples collected in 2009 and 2011, representing periods before and after routine PCV7 use (*12*) (Technical Appendix).

Nasopharyngeal samples were classified according to their collection time surrounding ARIs: peri-ARI periods included pre-ARI (8–14 or 1–7 days before an ARI) and post-ARI (1–7 or 8–14 days after an ARI). Samples outside these periods were considered non-ARI samples. We compared log-transformed pneumococcal nasopharyngeal densities of samples from ARI, peri-ARI, and non-ARI periods by using multivariable quantile regression with robust SEs and adjusting for relevant covariates. 

In secondary analyses, we assessed the role of respiratory viruses on pneumococcal density in children with ARIs. Because detection of nonrhinovirus respiratory viruses in nasal swabs was infrequent, we grouped samples into 4 distinct groups: rhinovirus only, rhinovirus and other viruses, other viruses only, and negative for any viruses.

We examined the role of pneumococcal acquisition on pneumococcal density using pneumococci-positive nasopharyngeal samples from children who had a sample collected within the preceding 60 days. Samples were categorized as 1) new colonization if the prior sample was negative, 2) serotype persistence if the prior sample was the same serotype, and 3) serotype replacement if the prior sample was a different serotype. If either serotype was nontypeable or unknown, the pattern was considered undetermined.

We assessed 3,579 nasopharyngeal samples from 833 children: 450 (12.6%) were collected during ARIs, 956 (26.7%) during peri-ARI periods, and 2,173 (57.8%) during non-ARI periods. The median age was 1.39 years. The median duration for ARIs was 8 days (interquartile range [IQR] 5–13 days). According to IMCI-WHO criteria, 33 samples were associated with pneumonia or severe pneumonia (*13*) (Table).

**Table:** Demographic characteristics for children from whom nasopharyngeal swab samples were collected during different periods surrounding ARIs, Peru, 2009–2011*

Characteristic	Period of sample collection						Total, N = 3,579
	Non-ARI, n = 2,173	Pre-ARI		Current ARI, n = 450	Post-ARI		
		8–14 days, n = 211	1–7 days, n = 222		1–7 days, n = 332	8–14 days, n = 191	
No. children†	765	186	189	320	262	172	833
Demographics							
Median age, y, at sample collection	1.45	1.36	1.37	1.23	1.25	1.37	1.39
Male	50.5	56.4	52.3	52.9	49.4	48.2	51.1
Attend daycare equivalent	7.8	7.1	8.6	4.2	6.6	5.2	7.1
Patient’s home							
Traditional stoves for cooking	63.1	73.5	63.5	61.1	68.7	62.8	64.0
Running water	24.7	17.5	17.6	24.0	18.7	27.2	23.3
Sewer or septic tank	21.1	18.5	18.5	20.4	22.3	22.5	20.9
Electricity	42.3	34.6	37.4	38.7	40.7	41.9	40.9
Season and year at sample collection							
Fall 2009	4.4	8.1	6.8	9.8	6.0	6.8	5.7
Winter 2009	19.3	25.1	27.5	30.0	23.2	25.7	22.2
Spring 2009	23.0	19.0	17.1	19.3	23.2	19.9	21.8
Fall 2011	24.6	25.1	24.3	21.6	22.6	25.1	24.1
Winter 2011	28.7	22.8	24.3	19.3	25.0	22.5	26.2
Altitude, m, of residence							
1,976–2,321, quartile 1	25.4	26.5	23.9	23.8	24.7	26.7	25.1
2,322–2,644, quartile 2	25.1	23.2	24.3	23.8	25.6	26.7	24.9
2,645–2,861, quartile 3	24.6	19.9	25.7	29.3	24.7	25.7	25.0
2,862–3,803, quartile 4	24.9	30.3	26.1	23.1	25.0	20.9	24.9
*Data are %, except for no. children. ARIs, acute respiratory illnesses; non-ARI, a period outside the pre-ARI, current ARI, and post-ARI periods. †No. children who contributed samples during each period.							

Overall, 36.7% of nasopharyngeal samples were from children who had received >2 PCV7 doses and were considered vaccinated. Approximately 5.0% of samples were from children who had received aminopenicillins, cotrimoxazole, chloramphenicol, or furazolidone within the 7 days preceding sample collection.

Quantitative PCR detected *S. pneumoniae* in 68.9% of nasopharyngeal samples; 78.9% of ARI and 65.3% of non-ARI samples were positive (p = 0.06). Unadjusted log-transformed pneumococcal densities varied by ARI periods (Technical Appendix).

**Figure 1:**
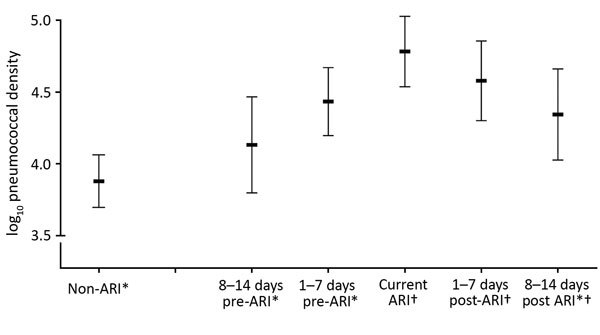
Estimated median pneumococcal densities with 95% CIs (vertical bars) by acute respiratory illness (ARI) period. Estimates derived from a quantile regression model that accounted for sex, age, daycare attendance, electricity, water supply, housing materials, kitchen type, smokers at home, vaccination, antimicrobial drug use, season, and altitude of residence. Asterisk indicates significantly different from ARI samples; dagger indicates significantly different from non-ARI samples.

Adjusted analyses showed that densities peaked during ARIs. In post hoc adjusted comparisons, densities were higher during the 1–7 days pre-ARI (p<0.0001), ARI (p<0.0001), 1–7 days post-ARI (p<0.0001), and 8–14 days post-ARI (p = 0.007) than during the non-ARI period (Figure 1).

Of 450 ARI nasopharyngeal samples, 435 (97%) had corresponding nasal swab samples available for identification of respiratory viruses; 299 (68.7%) tested positive for at least 1 virus. Rhinovirus, which was detected in 44.6% (194/435) of samples, was the most common virus (Technical Appendix). The median log-transformed pneumococcal densities of 299 virus-positive samples and 136 virus-negative samples were not significantly different (4.73 vs 3.94, respectively; adjusted p = 0.06).

**Figure 2:**
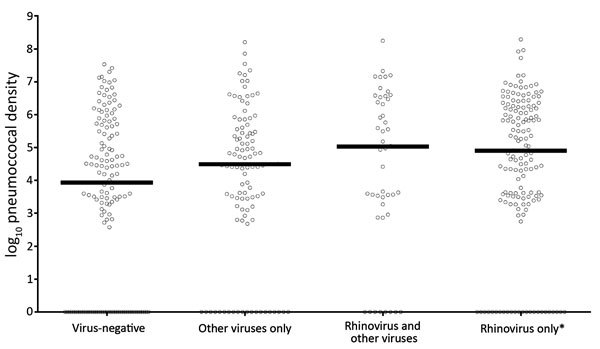
Pneumococcal densities of current acute respiratory illness samples subdivided by reverse transcription PCR detection of respiratory viruses. Each circle represents a single bacterial density measurement. The median for the samples of each subgroup is represented by a gray horizontal line. Asterisk indicates significantly different from virus-negative samples.

During ARI, the median log-transformed pneumococcal densities varied among virus groups: virus-negative (3.94, IQR 0.00–5.67; n = 136), nonrhinovirus (4.49, IQR 3.12–5.48; n = 105), rhinovirus-only (4.91, IQR 3.43–6.23; n = 147), and rhinovirus detected with other viruses samples (5.03, IQR 3.28–6.53; n = 47). In multivariable analyses, the only significant difference was between rhinovirus-only and virus-negative samples (p = 0.02) (Figure 2).

For the colonization patterns assessment, 2,479 (69.3%) nasopharyngeal samples had another sample collected <60 days before the current sample; the median time between samples was 28 days. The median log-transformed pneumococcal densities among samples that represented new colonizations (5.14, IQR 3.56–6.24; n = 411), serotype replacement (5.49, IQR 4.53–6.44; n = 322), and serotype persistence (5.79, IQR 4.82–6.47; n = 489) were compared. In multivariable analysis, serotype-replacement (p = 0.005) and serotype-persistence (p = 0.0003) samples had higher density than new colonization samples. The difference between serotype replacement and serotype persistence was not significant (p = 0.2).

## Conclusions

Our findings demonstrate a dynamic evolution of pneumococcal densities before, during, and after ARI episodes among young children. We observed a gradual increase in pneumococcal density leading up to an ARI episode, peak density during symptomatic ARI, and a decrease in density post-ARI to levels similar to those in baseline non-ARI periods.

Our observations of higher densities during ARI than non-ARI episodes align with those in studies from Vietnam and South Africa (*14*,*15*) and complement those assessments by illustrating the dynamic evolution of pneumococcal densities and the role of virus co-infections and pneumococcal colonization patterns. Unlike other studies that focused on hospitalized children, our community-based study showed relatively modest variations in nasopharyngeal pneumococcal density. 

Rhinovirus detection was associated with increased pneumococcal density during ARI. Although we observed an even higher median pneumococcal density in samples co-infected with rhinovirus and other respiratory viruses, the number of observations was small and statistical power to demonstrate significant differences was limited.

Compared with new colonization in our study, serotype persistence and replacement were associated with higher pneumococcal density. Because many new colonizations might ultimately succumb to host mechanisms and fail to establish stable colonization (*2*), the observed lower densities might reflect a decline of pneumococcal populations as clearance evolved. Nevertheless, although statistically significant, the differences in density were relatively modest, and we cannot establish the precise time of colonization or clearance in our samples.

Our study has several limitations. ARI identification depended on the presence of cough or fever, which are subjective but widely used for routine ARI surveillance (*5*–*7*). Because our study used household-based rather than health facility–based surveillance, severe disease was infrequent, precluding detailed assessments of disease severity. Due to small numbers, we could not study serotype-specific pneumococcal densities. In addition, because the study was conducted in rural communities of Peru, caution is warranted when extrapolating our findings to other settings.

Our findings demonstrated that, among young children, nasopharyngeal pneumococcal density started increasing before the onset of ARI symptoms, peaked during symptomatic ARI, and decreased after symptoms subsided. Rhinovirus co-infection, serotype persistence, and serotype replacement were associated with increased nasopharyngeal pneumococcal density. Nasopharyngeal pneumococcal density is dynamic surrounding ARI episodes and likely driven by complex virus–bacteria–host interactions.

Technical Appendix Expanded description of methods and results for a study of the role of nasopharyngeal pneumococcal density in the evolution of acute respiratory illnesses in young children, Peru, 2009–2011.
