# Supplementary material for: Nasopharyngeal Pneumococcal Density and Evolution of Acute Respiratory Illnesses in Young Children, Peru, 2009–2011
Source: Emerg Infect Dis. 2016 Nov;22(11):1996–9. doi: 10.3201/eid2211.160902 (PMC5088003; doi:10.3201/eid2211.160902)
Supplement: Technical Appendix — Expanded description of methods and results for a study of the role of nasopharyngeal pneumococcal density in the evolution of acute respiratory illnesses in young children, Peru, 2009–2011. [file 16-0902-Techapp-s1.pdf]

Article DOI: http://dx.doi.org/10.3201/eid2211.160902

Nasopharyngeal Pneumococcal Density and Evolution of Acute Respiratory Illnesses in Young Children, Peru, 2009–2011

Technical Appendix

Expanded Description of Methods

Study Population

Our prospective cohort study (RESPIRA-PERU) (*1*,*2*) was designed to examine the epidemiology and etiology of acute respiratory illness (ARI) in children <3 years old in the District of San Marcos, Cajamarca, Peru. The local population is mostly low income, living in rural communities with limited access to healthcare (*1*). The 7-valent pneumococcal conjugate vaccine (PCV7) was initially introduced into the communities in late 2009, and uptake slowly increased throughout the study period. This study was approved by the institutional review boards of Vanderbilt University (Nashville, TN, USA) and the Instituto de Investigacion Nutricional (Lima, Peru).

Weekly Household Visits

In the RESPIRA-PERU study, local field workers were trained to interview parents about respiratory signs and symptoms of respiratory illness based on the Integrated Management of Childhood Illness (IMCI-WHO) protocol (*3*,*4*). For this study, an ARI episode was defined as the period of time a child had either cough or fever (*5*,*6*). If ill at the time of the weekly household visit, the presence of IMCI-WHO pneumonia danger signs (inability to drink or breastfeed, persistent vomiting, convulsions, lethargy, unconsciousness, stridor, severe malnutrition) or signs of lower respiratory tract infection (tachypnea, audible wheezing, chest retractions, grunting, nasal flaring, stridor, or cyanosis) was assessed (*4*,*5*).

Respiratory Samples

Nasal swabs were collected for each ARI episode (through 7 days after symptom resolution), and processed as previously described (*6*,*7*). Samples were shipped to Vanderbilt University for detection of influenza viruses, respiratory syncytial virus, human metapneumovirus, rhinovirus, adenovirus, and parainfluenza viruses by real-time reverse transcription PCR (RT–PCR) (*6*,*8*–*10*). Monthly nasopharyngeal (NP) samples were collected according to WHO guidelines (*11*,*12*), transported in 1 mL of skim milk-tryptone-glucose-glycerine (STGG) medium and frozen in the media at −70°C. Analyses at Emory University (Atlanta, GA< USA) included culture and DNA extraction from *Streptococcus pneumoniae* isolates and NP specimens using a QIAamp DNA Mini Kit (QIAGEN, Valencia, CA). Colonization density was determined using a targeted *lytA* gene real-time quantitative PCR (qPCR)(*13*,*14*). Pneumococcal colonization density (CFU/mL) was quantified using purified genomic DNA from *S. pneumoniae* reference strain TIGR4 and serially diluted 10-fold to prepare standards (4 × 10° to 4 × 10^6 CFU) (*14*). Standards were run along with DNA from NP samples in a CFX96 real-time PCR detection system (Bio-Rad, Hercules, CA) using the software Bio-Rad CFX manager (*14*). Pneumococcal serotyping was performed by multiplex PCR (*13*,*15*,*16*). We used samples collected in 2009 and 2011, representing the time periods before and after routine use of PCV7 in the study communities, respectively (*17*).

ARI and Peri-ARI Periods

The duration of an ARI episode was defined as the number of days a child had either cough or fever (*5*,*6*). We defined peri-ARI periods including 8–14 days pre-ARI, 1–7 days pre-ARI, 1–7 days post-ARI and 8–14 days post-ARI. Samples collected outside the above categories were classified as non-ARI. When a sample met criteria for >1 category because it was collected between 2 ARIs, it was classified relative to the closest ARI.

Patterns of Pneumococcal Serotype Acquisition

A secondary analysis included all pneumococcal-positive samples in which there was a previous NP sample within 60 days. When the previous sample was qPCR negative, the current sample was classified as new colonization. When the previous sample was qPCR positive, the current sample was classified as persistence if the serotype was the same, or as replacement if a different serotype was detected. If either serotype was nontypeable or unknown, then the pattern was considered undetermined. Unknown refers to those samples for which the serotyping

reactions were inconclusive. Some samples that are *lytA* positive may contain insufficient pneumococcal material for the multiplex PCR reactions to test positively for any serotype.

Statistical Analyses

We compared pneumococcal NP densities during ARI, peri-ARI, and non-ARI periods. Bacterial densities were presented on a logarithmic scale. To retain the samples with zero density, we applied a $\log_{10}(x+1)$ transformation, where x represents the measured density. The median density was chosen as the most appropriate measure of central tendency as it is more robust to extreme values. Due to the persistent non-normal distribution of the transformed values, we used multivariable quantile regression to investigate the relationship between the log-transformed pneumococcal densities, periods of observation and relevant covariates. These covariates included sex, age, daycare attendance, electricity, water supply, housing materials, kitchen type, smokers at home, vaccination, antimicrobial drug use, season, and altitude. We used a restricted cubic spline function to allow for nonlinear effects of age in the model. Since a child could contribute >1 sample for our analyses, we accounted for this correlation of observations by calculating robust SEs using the Huber-White Sandwich variance estimator. The median pneumococcal densities for comparison groups were calculated using adjusted predictions from the respective quantile regression models, while integrating the influence of other model parameters. For post hoc comparisons of adjusted densities between the non-ARI and the ARI periods, we conservatively applied a Bonferroni correction to the type I error (5 comparisons, and a corrected $p<0.01$ to define statistical significance).

We conducted secondary analyses to assess the role of respiratory viruses on pneumococcal density, restricting the analysis to samples collected during ARI or post-ARI periods. Given that rhinoviruses were commonly detected but other viruses were less frequently detected, samples were classified into 4 mutually exclusive groups: 1) positive for rhinovirus only, 2) positive for rhinovirus and other viruses, 3) positive for other viruses only; and 4) negative for any viruses. Another secondary analysis examined the role of the previously described patterns of pneumococcal serotype acquisition on pneumococcal density. For all secondary analyses, we used multivariate quantile regression models to account for covariates. Stata® version 14.0 was used for all statistical analyses (StataCorp, College Station, TX, USA).

Expanded Description of Results

Summary of Enrollment and Surveillance in Parent RESPIRA-Peru Study

Enrollment and surveillance in the RESPIRA-Peru study have been described elsewhere (*1*). In brief, during May 2009–September 2011, a total of 892 children were enrolled, 55,661 household visits were scheduled, 89% were executed (i.e., field workers reached their target households), and 79% were successfully completed (i.e., information was collected during the visit). A total of 4,655 nasal and 10,722 NP swabs were collected. Collection of NP swabs was completed in 88% of scheduled collections.

Additional Details on Pneumococcal Density Assessments

The unadjusted log-transformed pneumococcal densities are shown for each period in Technical Appendix Figure. In unadjusted analysis, the median NP density during the non-ARI periods (3.71, IQR 0.00–5.59) was statistically lower than the 1–7 days pre-ARI (4.42, IQR 2.72–5.86; $p<0.001$), the ARI period (4.49, IQR 3.04–5.95; $p<0.001$), and 1–7 days post-ARI (4.48, IQR 0.00–5.79; $p = 0.002$) periods.

In the multivariable quantile regression model that examined the association between ARI study periods and pneumococcal density, season was significantly associated with median pneumococcal density. Spring (October, November) 2009, Fall (May, June) 2011, and Winter (July, August, September) 2011 were associated with elevated density compared to Fall 2009 and Winter 2009. Housing characteristics (including running water, electricity, and sewage system), PCV7 vaccination, health facility visits, antimicrobial drug use and the presence of smokers in the home did not have statistically significant associations with median pneumococcal density.

References

1. Grijalva CG, Griffin MR, Edwards KM, Williams JV, Gil AI, Verastegui H, et al. Cohort profile: the study of respiratory pathogens in Andean children. Int J Epidemiol. 2014;43:1021–30. PubMed http://dx.doi.org/10.1093/ije/dyt065

2. Budge PJ, Griffin MR, Edwards KM, Williams JV, Verastegui H, Hartinger SM, et al.; RESPIRA-PERU Group. A household-based study of acute viral respiratory illnesses in Andean children. Pediatr Infect Dis J. 2014;33:443–7. PubMed http://dx.doi.org/10.1097/INF.0000000000000135

3. WHO Division of Child Health and Development. Integrated management of childhood illness: conclusions. Bull World Health Organ. 1997;75(Suppl 1):119–28. PubMed

4. Gove S; The WHO Working Group on Guidelines for Integrated Management of the Sick Child. Integrated management of childhood illness by outpatient health workers: technical basis and overview. Bull World Health Organ. 1997;75(Suppl 1):7–24. PubMed

5. Lanata CF, Rudan I, Boschi-Pinto C, Tomaskovic L, Cherian T, Weber M, et al. Methodological and quality issues in epidemiological studies of acute lower respiratory infections in children in developing countries. Int J Epidemiol. 2004;33:1362–72. PubMed http://dx.doi.org/10.1093/ije/dyh229

6. Poehling KA, Edwards KM, Weinberg GA, Szilagyi P, Staat MA, Iwane MK, et al.; New Vaccine Surveillance Network. The underrecognized burden of influenza in young children. N Engl J Med. 2006;355:31–40. PubMed http://dx.doi.org/10.1056/NEJMoa054869

7. Griffin MR, Walker FJ, Iwane MK, Weinberg GA, Staat MA, Erdman DD; New Vaccine Surveillance Network Study Group. Epidemiology of respiratory infections in young children: insights from the new vaccine surveillance network. Pediatr Infect Dis J. 2004;23(Suppl):S188–92. PubMed http://dx.doi.org/10.1097/01.inf.0000144660.53024.64

8. Kodani M, Yang G, Conklin LM, Travis TC, Whitney CG, Anderson LJ, et al. Application of TaqMan low-density arrays for simultaneous detection of multiple respiratory pathogens. J Clin Microbiol. 2011;49:2175–82. PubMed http://dx.doi.org/10.1128/JCM.02270-10

9. Klemenc J, Asad Ali S, Johnson M, Tollefson SJ, Talbot HK, Hartert TV, et al. Real-time reverse transcriptase PCR assay for improved detection of human metapneumovirus. J Clin Virol. 2012;54:371–5. PubMed http://dx.doi.org/10.1016/j.jcv.2012.05.005

10. Lu X, Holloway B, Dare RK, Kuypers J, Yagi S, Williams JV, et al. Real-time reverse transcription–PCR assay for comprehensive detection of human rhinoviruses. J Clin Microbiol. 2008;46:533–9. PubMed http://dx.doi.org/10.1128/JCM.01739-07

11. O’Brien KL, Nohynek H; World Health Organization Pneumococcal Vaccine Trials Carriage Working Group. Report from a WHO Working Group: standard method for detecting upper respiratory carriage of *Streptococcus pneumoniae*. Pediatr Infect Dis J. 2003;22:e1–11. PubMed http://dx.doi.org/10.1097/01.inf.0000049347.42983.77

12. Satzke C, Turner P, Virolainen-Julkunen A, Adrian PV, Antonio M, Hare KM, et al.; WHO Pneumococcal Carriage Working Group. Standard method for detecting upper respiratory carriage of *Streptococcus pneumoniae*: updated recommendations from the World Health Organization Pneumococcal Carriage Working Group. Vaccine. 2013;32:165–79. PubMed http://dx.doi.org/10.1016/j.vaccine.2013.08.062

13. Klugman KP, Madhi SA, Albrich WC. Novel approaches to the identification of *Streptococcus pneumoniae* as the cause of community-acquired pneumonia. Clin Infect Dis. 2008;47(Suppl 3):S202–6. PubMed http://dx.doi.org/10.1086/591405

14. Chien YW, Vidal JE, Grijalva CG, Bozio C, Edwards KM, Williams JV, et al. Density interactions among *Streptococcus pneumoniae*, *Haemophilus influenzae* and *Staphylococcus aureus* in the nasopharynx of young Peruvian children. Pediatr Infect Dis J. 2013;32:72–7. PubMed http://dx.doi.org/10.1097/INF.0b013e318270d850

15. Albrich WC, Madhi SA, Adrian PV, van Niekerk N, Mareletsi T, Cutland C, et al. Use of a rapid test of pneumococcal colonization density to diagnose pneumococcal pneumonia. Clin Infect Dis. 2012;54:601–9. PubMed http://dx.doi.org/10.1093/cid/cir859

16. Vernet G, Saha S, Satzke C, Burgess DH, Alderson M, Maisonneuve JF, et al. Laboratory-based diagnosis of pneumococcal pneumonia: state of the art and unmet needs. Clin Microbiol Infect. 2011;17(Suppl 3):1–13. PubMed http://dx.doi.org/10.1111/j.1469-0691.2011.03496.x

17. Hanke CR, Grijalva CG, Chochua S, Pletz MW, Hornberg C, Edwards KM, et al. Bacterial density, serotype distribution and antibiotic resistance of pneumococcal strains from the nasopharynx of Peruvian children before and after pneumococcal conjugate vaccine 7. Pediatr Infect Dis J. 2016;35:432–9. PubMed http://dx.doi.org/10.1097/INF.0000000000001030

Technical Appendix Table 1. Viral detections in nasal swab samples*

Virus	No.	Nasal swab samples, %
Rhinovirus	257	51.2
Adenovirus	84	16.7
Influenza	46	9.2
Parainfluenza 1–3	42	8.4
Respiratory syncytial virus	40	8.0
Human metapneumovirus	33	6.6

*Some samples had >1 virus detected.

Technical Appendix Table 2. Pneumococcal serotype detections in NP swab samples*

Serotype	No.	NP swab samples, %
1	4	0.2
10A	87	4.3
10F/10C/33C	14	0.7
11A	102	5.0
11A/11D	53	2.6
12F	2	0.1
13	51	2.5
14	49	2.4
15A	38	1.9
15A/15F	22	1.1
15B	37	1.8
15B/15C	31	1.5
15C	38	1.9
16F	15	0.7
17F	26	1.3
18A	1	0.1
18A/18B/18C	8	0.4
18A/18B/18C/18F	8	0.4
18C	9	0.4
19A	61	3.0
19F	159	7.8
2	1	0.1
20	6	0.3
21	16	0.8
22A	3	0.2
22F	16	0.8
22F/22A	8	0.4
23A	25	1.2
23B	57	2.8
23F	138	6.8
24A/24B/24F	6	0.3
25F	1	0.1
28A	7	0.3
3	29	1.4
31	6	0.3
33A/33F/37	8	0.4
33B	7	0.3
33B/33D	2	0.1
33F	10	0.5
34	22	1.1
35A	16	0.8
35A/35C/42	18	0.9
35B	29	1.4
35C	1	0.1
35F	27	1.3
35F/47F	21	1.0
38/25F	7	0.3
39	3	0.2
4	16	0.8
5	2	0.1
6A	42	2.1
6A/6B	51	2.5
6A/6B/6C	69	3.4
6B	111	5.4
6C	127	6.2
6D	5	0.2
7B/7C/40	11	0.5
7C	27	1.3
7F	5	0.2
8	3	0.2
9A	3	0.2
9A/9V	13	0.6
9L/9N	10	0.5
9N	1	0.1
9V	12	0.6
NT	229	11.2
Total	2,042	100

*When serotyping reactions were unable to differentiate between $\geq$2 serotypes, the multiple serotypes are reported. NP, nasopharyngeal.

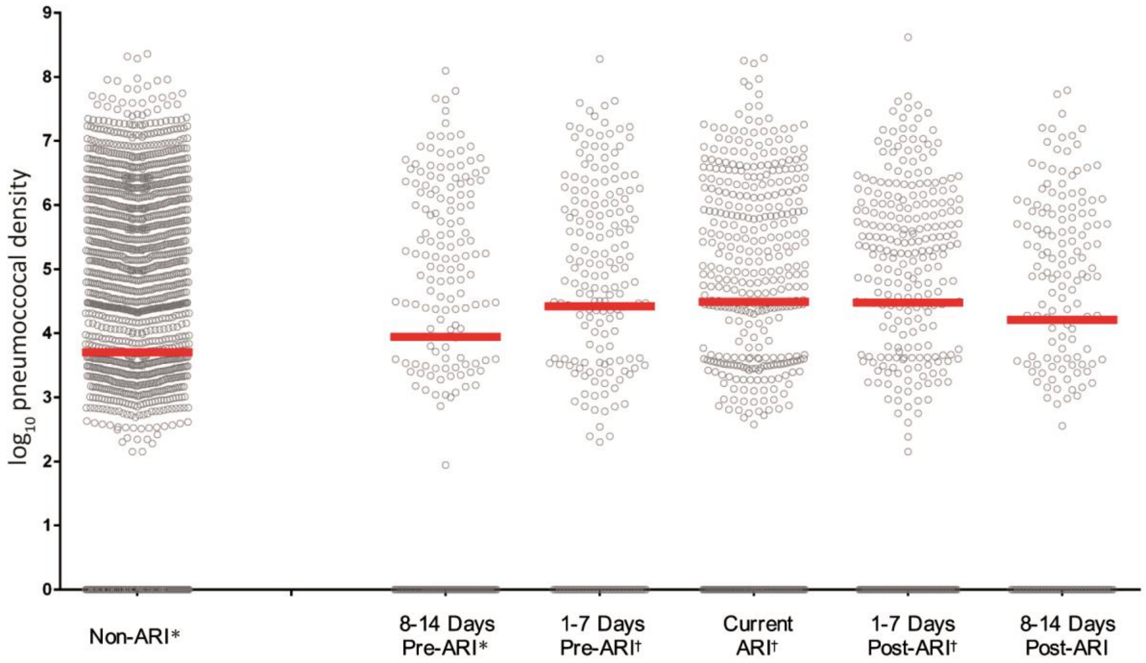

Technical Appendix Figure. Pneumococcal densities for each acute respiratory illness (ARI) period. Each circle represents a single bacterial density measurement. The median for the samples of each period is represented by a red line. *Significantly different from ARI samples. †Significantly different from non-ARI samples.
